# Super Aging in South Korea Unstoppable but Mitigatable: A Sub-National Scale Population Projection for Best Policy Planning

**DOI:** 10.1007/s40980-020-00061-8

**Published:** 2020-06-12

**Authors:** Kee Whan Kim, Oh Seok Kim

**Affiliations:** 1 Department of National Statistics, Korea University, Sejong, South Korea; 2 Department of Geography, Graduate School of Korea University, #633, Uncho-Useoun Hall, 145 Anam-ro, Seongbuk-gu, Seoul 02841, South Korea; 3 Department of Geography Education, College of Education, Korea University, Seoul, South Korea; 4 Institute of Future Land, Korea University, Seoul, South Korea

**Keywords:** Aging, Super-aged society, Population projection, Cohort component method, Migrant pool model, Time-series analysis

## Abstract

This research portrays the spatial and temporal progression of super-aging in regions throughout South Korea. Using a single-year population projection considering gross domestic migration, this research identifies which regions will shortly become a super-aged society. A cohort-component method with a migrant pool model is applied. The county-level national population registration data (2000–2018) are aggregated into 37 regions for the model run. In 2020, 16 rural regions will become super-aged societies. By 2029, all 37 regions, including the metropolitan areas, will join the group, with Sejong, the administrative capital, being the last to enter. In brief, the rural areas become super-aged earlier than the metropolitan areas, and within a decade, those 65 years old or older will make up the majority of the national population. Among all the metropolitan areas, Busan, the largest harbor city, will be the first to be super-aged in 2023. Sejong will experience the most radical change between 2020 and 2050. The research outcomes demonstrate that demographic changes in the rural and metropolitan areas are different; hence, the recent population policies, such as promoting fertility, may not work in the rural areas as they have already lost their population momentum due to the extreme and ongoing urbanization throughout the nation. The unstoppable aging will pose adverse effects on future citizens (who are mostly senior) both financially and medically. An increase in health care expenditure and a nationwide blood shortage for transfusion are anticipated, for example.

## 1 Introduction

### 1.1 Background

**Fig. 1:**
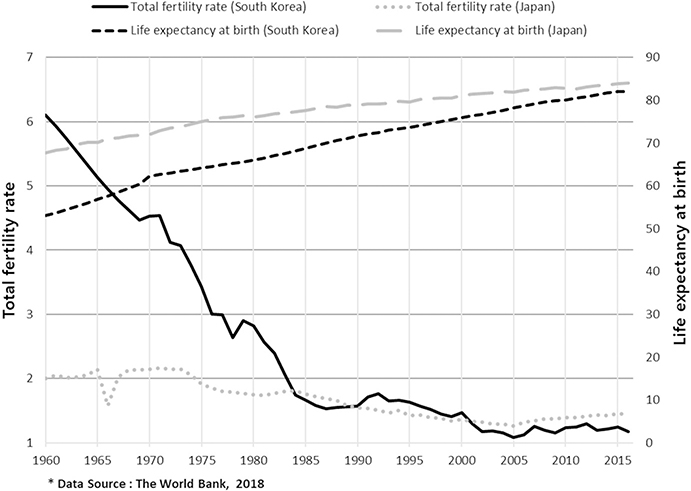
Change of total fertility rate (TFR) and life expectancy at birth (LEB) of South Korea and those of Japan between 1960 and 2016

Not many are aware of the fact that population aging in South Korea has been more severe than that in Japan. Although Japan’s aging may have “matured” earlier than South Korea’s, it is highly likely that South Korea will catch up with Japan in no time (Fig. 1). In 2017, South Korea had officially become an aged society, with more than 14 percent of its citizens 65 years old or older. It took only 17 years after South Korea became an aging society in 2000, while Japan took 24 years for the same transition (Chosun 2017). According to the World Health Organization, when an aging rate exceeds 7%, 14%, and 21% it is entitled “aging society,” “aged society,” or “super-aged society,” respectively; “aging rate” is defined as the proportion of a society’s population aged 65 or older. In addition, South Korea has been a member of the lowest-low fertility group, its total fertility rate (TFR) at or below 1.3, since 2002 (Choe and Retherford 2009; Whang and Choi 2015). In 2018, its TFR reached 0.98, which is the world’s lowest record (Bloomberg 2019; Statistics Korea 2019). At present, the country’s life expectancy at birth (LEB) is ranked 6th among 183 countries, while female LEB is ranked 3rd (OECD 2019). A recent study published in *The Lancet* foresees that South Korea will have the longest LEB (both male and female LEB ranked 1st) in 2030 among 35 developed countries (Kontis et al. 2017).

Aging in itself is a societal problem that is difficult to cope with, in part because it takes much time to resolve how to mitigate and adapt to such aging. In this context, constructing a spatially and temporally explicit population projection is necessary so that the society can adequately cope with the situation. Given the urgency, it is ideal to have a single-year population projection over 5 years, primarily because aging in South Korea rapidly evolves. Moreover, it is more helpful to have a population projection outcome at a finer spatial scale than a province or national level.

### 1.2 Literature Review

In generating population projections, two methods are commonly employed: (1) the cohort component method (hereafter, the component method) and (2) the cohort change ratios (hereafter, the ratio method). The former is regarded as the traditional “textbook” approach (Baker et al. 2017). This approach models various demographic components, namely births, deaths, and migrants (hence, a cohort *component* method), in a non-linear fashion based on the demographic balancing equation. That is why the method requires detailed and often extensive population datasets, which naturally consume many computing resources and require numerous statistical analyses. The component method is suitable for a nationwide population projection employing a long forecast horizon and is also capable of modeling gross migration and generating single-year projections. The latter ratio method is basically the Hamilton-Perry model (Smith et al. 2002) and is considered state-of-the-art due to its simple linear, yet transparent, logic allowing diverse applications with minimal data requirements. Usually, collecting and managing population data, such as decennial census and administrative record, are costly; hence, developing/least developed nations or poor municipalities are not readily equipped with such data. In these cases, applying a ratio method is ideal; therefore, the method has been frequently used over different countries at various geographical scales, including small-area forecasting (Baker et al. 2017). It is important to note that one method is not necessarily better than the other as each method has its own merits, so a selection between the existing methods should be determined based on the purpose of a population projection.

Both methods have been applied to case studies in South Korea. Statistics Korea—the government agency responsible for generating national population and household projections, conducting censuses, and collecting and fusing various types of national datasets—has frequently used the component method to generate single-year population projections on a regular basis. The agency’s population projections are provided at two geographical levels: the national and provincial levels. The former employs a lower spatial resolution (one national level), a longer time horizon (50 years), and international net migration (Statistics Korea 2019). In contrast, the latter employs a higher spatial resolution (17 provincial levels), a shorter time horizon (30 years), but no international migration (Statistics Korea 2017). Although both projections are official and provide essential information bases for relevant national policies in South Korea, it is evident that they are limited in their spatial resolution and migration modeling.

Applications of the ratio method in South Korea are more regional and spatially explicit than those of the component method. Either applied in urban (Kim 2008) or rural areas (Chang, Bae, and Seol 2019; Min and Kim 2014), they generate fairly accurate county-level population projections for the immediate future and are used for regional planning. However, there are common limitations: the case studies only show 5-year projections and do not consider migration in modeling. Such exclusion is problematic when one is to project a distant future (e.g., more than 30 years), especially given the rapid pace of aging, depopulation, and active migration in South Korea.

In summary, if one wants to generate a single-year population projection of South Korea that employs a similar forecast horizon of the existing national population (e.g., 30 years), then it is logical to choose the component method over the ratio method because a ratio method’s forecast horizon is usually no longer than 20 years (Baker et al. 2017). Of course, taking the migration modeling into account, associated with a downscaled geographical setting (e.g., 37 subregions instead of 17 provinces), would be a plus.

Although modeling gross migration is considered ideal in literature, it has not always been employed by a component method due to the challenge in accessing its data and estimating migration rates. Characteristically, modeling gross migration requires more data than modeling net migration, and such data availability is not always guaranteed. In estimating rates of in- and out-migration, such rates should be variable enough to factor in different population sizes, geographies, speed of change, etc. and yet must be stable enough to yield a reasonable and not too extreme outcome. Such a challenge appears to be valid for both multiregional and migrant pool models when modeling gross migration (Dion 2017; Rogers 2008; Wilson and Bell 2004).

In the case of South Korea, gross migration has been modeled in two ways. First, it was modeled in a multiregional context, a method subsequently criticized by Cho and Lee (2011) for employing net (not gross) migration. Statistics Korea then introduced fixed rates of origin–destination migration to take gross migration into account. Later on, this approach was replaced by Feeney’s destination population-weighted model because the shortcomings of estimating migration rates based on fixed (not variant) transition probabilities were pointed out by Plane (1993). Feeney’s model not only considers transition probabilities that change over time but also factors in population sizes of origins and destinations (Feeney 1973; Wilson and Bell 2004). The rationale behind the model is to incorporate both the magnitude of migrant sources and destination attractiveness, where these can vary over time (Statistics Korea 2017). A recent Canadian case study exemplified that such an approach was useful (Dion 2017).

Second, a novel migrant pool model was proposed by Lee et al. (2017) because the authors thought Feeny’s method was limited in dealing with numerous geographical areas, especially when they are downscaled. In other words, it consumes too much time and computation resources to process demographic data for a multiregional setting. Their approach was the first attempt to test a migrant pool model in South Korea, while similar types had been applied in the United States (US Census Bureau. 1967), European Union and the United Kingdom (Wilson and Bell 2004). The model structure and rationale are described as follows: First, the number of out-migrants of each region is estimated, and then the data for all out-migrants from all regions are stored in one virtual pool (hence, a migrant *pool* model). Second, from the pool, potential in-migrants are accounted for, and the quantities are allocated back to the regions (hence, in-migrants). The numbers of out- and in-migrants are determined based on the corresponding migration rates that are empirically and statistically estimated. Occasionally, population sizes of destinations can be incorporated in tandem when allocating the in-migrants to the destinations (Wilson and Bell 2004), but Lee et al. (2017) did not consider the population sizes for their Korean case study.

### 1.3 Research Questions

The purpose of this research is to project a sub-national aging population for the year 2050 so that one can learn how rapidly such aging would progress and vary over regions when the current situation remains as it is (i.e., business-as-usual). Knowing the size and structure of the future aging population and its sub-national placement is vital to relevant national and regional policies in South Korea. However, the current population projections lack geographical explicitness; therefore, it is difficult to design a well-targeted policy at a sub-national level. In this research, we present a new population projection that yields more spatially disaggregated outcomes compared to the exiting population projections of South Korea. The research questions are as follows:

1. Which region(s) will become a member of the super-aged society in 2020?
2. Which region(s) will be the last to join the super-aged society, and when?
3. Which metropolitan area will be the first to join the super-aged society, and when?
4. Which region(s) will experience the most drastic and the slowest aging between 2020 and 2050?

By answering these questions, we intend to foresee how the South Korean population would look when the lowest-low fertility and super-aged society evolves in a business-as-usual fashion. The future will be very different from when South Korea experienced the baby boom in the 1950s, right after the Korea War. Being equipped with a reliable population projection is, therefore, indispensable in dealing with any uncertainty in the future. Of course, no major war between South and North Korea, as well as no other severe catastrophe, is assumed in this study.

## 2 Data and Methods

### 2.1 Data

Administrative records showing the national population registration for the period between 2000 and 2018 were acquired from the Korean Statistical Information Service (http://kosis.kr/eng/). South Korea has generated and maintained quality population data for decades, and the data are free of charge, available for everyone, and downloadable in multiple formats. The record portrays the most recent population structure, such as age and sex, at a county level, every year, and it serves as a basis for voter registration, school enrollment, military conscription, etc. The record also comes with a series of geographic information systems (GIS) layers. Thus, the yearly administrative record data not only can be mapped using GIS but also can support the generation of a single-year population projection.

For the model run, South Korea is divided into 37 parts. That division is designed to include one larger hub city and multiple smaller satellite cities and/or counties closely connected to the hub city. Such division is based on the demographic microdata that show daily commute of the nation at the county level. In this way, it is expected that the newly data-driven divisions would represent people’s livelihood more realistically than formal, rigid administrative districts (Statistics Korea 2007). We used the recently updated divisions with newer data.

Accordingly, the county-level population data are aggregated into the 37 regions. The data are pre-processed to produce secondary datasets regarding fertility (e.g., period fertility rates and age-specific birth rates), mortality (e.g., life tables and age-specific log mortality rates), and migration (e.g., gross domestic migration rates).

International migration is not considered in this research because it was difficult to find relevant data. Our primary purpose is to enhance the spatial resolution of population projections for the South Korean case. It is vital, therefore, for us to employ 37 smaller geographical divisions (instead of 17 larger divisions) with the support of a novel migrant pool model. Otherwise, our work would show little difference compared to the existing work by the government. As aforementioned, even Statistics Korea (2017) was not able to factor in an international migration component for its lower resolution work.

### 2.2 Methods

#### 2.2.1 Cohort Component Method

A single-year cohort component method considering gross domestic migration (via a migrant pool model) is applied to generate age-, sex-, and region-specific population projections for the year 2050 (32-year projection horizon) at the sub-national level. The projection horizon is determined based on the national population projection horizon by Statistics Korea. The agency used the 30-year projection horizon for the sub-national projection for the 17 metropolitans and provinces (Statistics Korea 2017) and used the 50-year projection horizon for one national scale projection (Statistics Korea 2019). As Statistics Korea (2017) utilized data from 2001 to 2015 for the sub-national projection with the 30-year projection horizon, we thought it was reasonable to use data from 2000 to 2018. The projection is computed based on the demographic balancing equation, where the computation heavily relies on statistical extrapolation methods, such as autoregressive integrated moving average models or random walk with drift models.

#### 2.2.2 Fertility

A generalized log gamma distribution (GLG) model is employed to estimate and project age-specific fertility rates (ASFRs). The GLG model is constituted by four parameters and is expressed as follows:

(1) $$f(x)=\frac{C|\lambda |}{b\Gamma \left(\frac{1}{\lambda^{2}}\right)}{\left(\frac{1}{\lambda^{2}}\right)}^{\lambda^{-2}}\exp\left[\frac{1}{\lambda }\left(\frac{x-u}{b}\right)-\frac{1}{\lambda^{2}}\exp\left(\lambda \left(\frac{x-u}{b}\right)\right)\right]$$

where *f*(*x*) indicates the ASFR, *C* refers to the level of giving birth (0 ≤ *C* ≤ 1), *u* demonstrates the average age of women when giving birth (15 ≤ *C* ≤ 49), *b* shows the standard deviation of the average age of women when giving birth (*b* > 0), and *λ* is a constant value indicating birth order (−∞ ≤ *λ* ≤ ∞, *λ* ≠ 0).

The GLG model is a generalized version of the Coale-McNeil nuptiality model, which was based on Swedish data. Its generalized property makes the GLG model flexible enough to be applicable to diverse countries, e.g., South Korea or Japan, as the model was designed to factor in a country’s unique situation (Kaneko 2003). Given the South Korean case, the strengths of the GLG model are mainly twofold: First, the model parametrizes birth order, which is directly affected by any national population policies: namely government subsidy, parental leave, pension, etc. As South Korea has introduced diverse policy instruments, parametrizing the birth order supports a higher goodness-of-fit. Second, the GLG model utilizes period fertility rate (PFR) data, instead of cohort fertility rates (CFR). By nature, a CFR cannot be fully calculated until a cohort completes its lifetime, and it is uncertain how long the cohort will survive. That is, the CFR data have a fundamental data quality issue. Unlike the CFR data, however, the PFR, which is basically an annual fertility rate, does not have such a data quality issue, hence supports a better result for the South Korean situation (Young and Kim 2011).

The NLP (Non-Linear Programming) Procedure of SAS v9.2 is used for fitting the GLG model. For modeling the temporal trend of *C*, an autoregressive integrated moving average (ARIMA) model is applied because unlike the other two parameters, the recent trend of fluctuation is likely to affect the long-term projection of the parameter estimate; the ARIMA Procedure of SAS/ETS is used for the model fitting. For modeling the temporal trends of *u* and *b*, time-series regression models are applied because the pace of these parameter estimates’ change is relatively slow; the SYSLIN Procedure of SAS/ETS is used for the model fitting.

#### 2.2.3 Mortality

The Heligman and Pollard (HP) model is used to estimate age-specific log mortality rates (ASMRs), and the model is constituted by three terms, which is expressed as follows:

(2) $$\frac{q_{x}}{p^{x}}=A^{{\left(x+B\right)}^{C}}+D\exp\left(-E{\left(\ln\left(\frac{x}{F}\right)\right)}^{2}\right)+GH^{x}$$

where *q*^*x*^ indicates an ASMR of a person at age *x*, *p*^*x*^ is 1 − *q^x^*; $A^{{\left(x+B\right)}^{C}}$ demonstrates a declining tendency of mortality of early childhood years as children adapt to their new environments and gain immunity over time (*A* means the level of mortality, *B* refers to an age displacement to account for infant mortality, and *C* measures the pace of mortality decline); $De^{-E{\left(\ln(x)-\ln(F)\right)}^{2}}$ reflects accident mortality (*D* indicates the severity, and *E* and *F* show spread and location, respectively); and *GH*^*x*^ depicts increasing mortality due to aging (*G* indicates the base level of mortality due to biological aging, and *H* refers to the rate of increase of such mortality) (Heligman and Pollard 1980).

The NLP Procedure is used for fitting the HP model. For modeling the temporal trends of the parameter estimates, random walk with drifts models and linear Holt-Winters exponential smoothing are used through SAS/ETS.

#### 2.2.4 Migration

A migrant pool model is used to dictate the gross migration. First, the out-migration rates by age and sex are calculated to provide projections of out-migrants from the 37 regions over the 32-year projection horizon. These numbers are then added up to yield the age and sex-specific “migrant pool,” indicating the potential in-migrants for the entire regions. After the in-migration rates are estimated by age and sex for each region, the migrant pool is proportionally allocated based on the given age and sex structure.

The equations estimating the rates of out- and in-migration are as follows:

(3) $$\log\left(M{\left(g,s\right)}_{i,t}\right)=\log\left(\frac{I{\left(g,s\right)}_{i,t}}{P{\left(g\right)}_{i,t}}\right)$$

where *M*(*g*, *s*)_*i,t*_ indicates a migration rate at region *i* in year *t* while *g* means sex and *s* denotes the migration type; *I*(*g*, *s*)_*i,t*_ refers to a number of people who migrated from or to region *i* in year *t* for each sex and migration type; and *P*(*g*)_*i,t*_ means the total population at region *i* in year *t. M*(*g*, *s*)_*i,t*_ is estimated using the following equation:

(4) $$\log\left(M{\left(g,s\right)}_{i,t}\right)=a_{i}+b_{i}k_{t}+\varepsilon_{i,t}$$

where *a*_*t*_ shows the region-specific (but time-invariant) trend of change in migration rate at region *i*; *b*_*i*_ refers to the speed of change in migration rate at region *i*; *k*_*t*_ denotes the time-variant change of migration rate in year *t*; and *ε*_*i,t*_ is the error term with zero mean and variance $\sigma_{\varepsilon }^{2}$. A singular value decomposition is applied to estimate the parameters, where the idea was originated from Lee and Carter (1992). Although the LC model is well known for its applications on mortality, this statistical model is technically applicable for estimating other kinds of rates due to its data-driven nature.

After estimating the sex-specific gross out-migration and in-migration rates for each region, the quantity is allocated based on the given age structure through the following equation:

(5) $$\hat{I}{\left(g,s\right)}_{i,j,t+1}=\left[\hat{M}{\left(g,s\right)}_{i,t+1}\times P{\left(g\right)}_{i,t}\right]\times \left[\frac{I{\left(g,s\right)}_{i,j,t}}{\sum_{j=0}^{100+}I{\left(g,s\right)}_{i,j,t}}\right]$$

where $\hat{I}{\left(g,s\right)}_{i,j,t+1}$ refers to a number of estimated migrants in region *i* whose age are *j* in year *t* + 1 while *g* means sex and *s* demonstrates the migration type; $\hat{M}{\left(g,s\right)}_{i,t+1}$ indicates the estimated gross migration rate at region *i* in year *t* + 1 that is calculated from the previous step; and *P*(*g*)_*i,t*_ means the total population at region *i* in year *t*.

## 3 Results

The purpose of the range of estimation and model runs is to dictate the future aging population of South Korea through a single-year cohort component method considering gross domestic migration (via a migrant pool model) by generating age-, sex-, and region-specific population projections for the year 2050 at the sub-provincial level. By doing so, one can see how the demographic structure of South Korea would look if the current trend remains as it is.

**Fig. 2:**
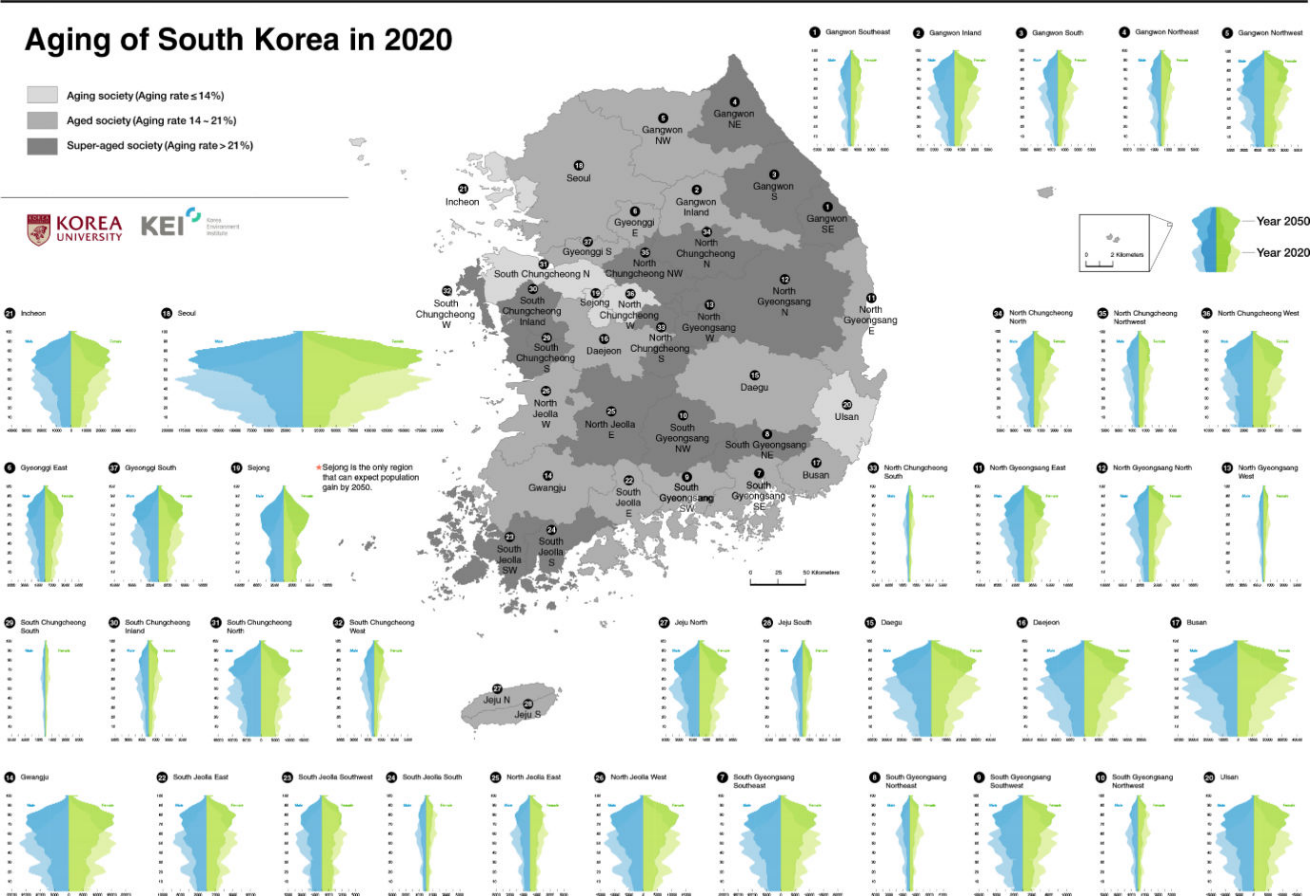
Aging, aged, and super-aged regions in 2020

In 2020, 16 regions out of 37 will become super-aged; the list of the regions is shown in Table 1 and mapped in Fig. 2, indicated by the darkest color. This result means that those 16 regions have at least 21% of their populations whose ages are equal to or older than 65. Aging is most severe (33.99%) in the region denoted as South Jeolla South—in terms of the absolute degree of aging rate (not necessarily the speed of aging)—followed by South Chungcheong South (33.46%) and South Gyeongsang Northwest (33.11%) (Table 1). Although these 16 regions are vast in area, as shown in Fig. 2, their total population is only worth 7.47% of the national population (3.78 million out of 50.61 million people in 2020). Implied by their small population sizes, the regions are mostly rural and remote. The average aging rate of the 16 regions is 24.27% in 2020.

**Fig. 3:**
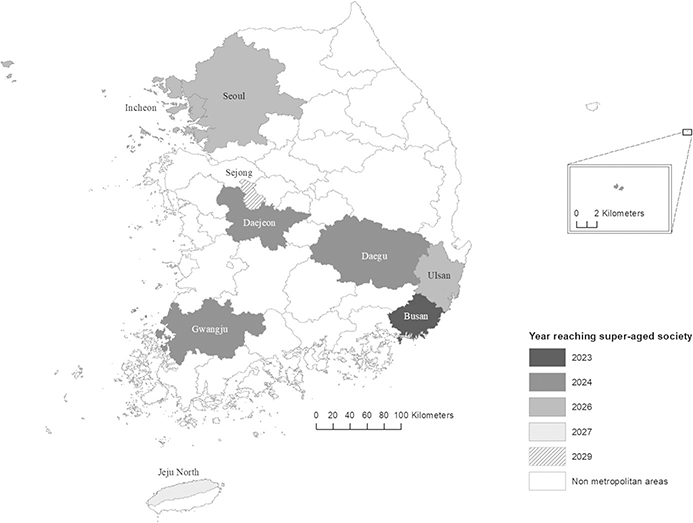
Aging of the metropolitan areas

By 2029, all 37 regions will join the super-aged society, with Sejong, the administrative capital of South Korea, being the last to enter. That is, South Korea will become super-aged both at the regional and national levels. Even though Sejong is the last region to become super-aged, it is only a decade apart from the rural and remote regions that already joined the super-aged society in 2020. This finding points out that other metropolitan areas will become super-aged earlier than Sejong, all within only a decade (Table 1 and Fig. 3).

Starting from Busan in 2023, all metropolitan areas will become super-aged by 2029. In the following year, Daejeon, Daegu, and Gwangju metropolitan areas will be super-aged. In 2026, Seoul, Incheon, and Ulsan metropolitan areas will join the group, followed by Jeju North and Sejong in 2028 and 2029, respectively (Fig. 3). In terms of population size, Jeju North and Sejong are not as significant as the other metropolitan areas, but they are essential urban hubs in the nation, so they are considered with others nonetheless. The total population of these nine regions is equivalent to 73.72% of the national population (while the total population of Seoul, the largest metropolitan and the capital region, is worth 40.48% of the national population), and the ratio will remain similar in 2050 (73.91%). As one can see from the result, South Korea is a highly urbanized nation; therefore, the aging in the metropolitan areas will directly shape the national aging in general.

By taking the difference between the aging rate in 2020 and that in 2050, the aging speeds among the regions are compared (Table 1). Sejong will go through the most drastic aging, followed by Incheon and Seoul. Given that Sejong is the “youngest” and Seoul is the largest metropolitan areas (while Incheon is the second largest harbor city that is adjacent to Seoul), one can easily foresee how massive the impact of their aging would be. The slowest aging, in contrast, is spatially concentrated in rural and remote areas. South Chungcheong South, in fact, shows a negative trend, followed by South Jeolla South and South Gyeongsang Northwest, where theirs are also negative. In short, aging in the metropolitan areas will be more drastic (both in terms of size and speed) than that in the rural and remote areas between 2020 and 2050. The aging in these rural and remote areas started earlier than the metropolitans and have evolved for a longer time period; hence, there is relatively less room for further aging, compared to the aging in metropolitan areas.

## 4 Discussion

### 4.1 Implications of Aging

Aging in itself is a common transition due to advanced medical treatment and health services (i.e., health and mortality transition). That is why aging is more often observed in developed countries than in developing and/or least-developed countries. Aging will slowly progress even with a constant fertility rate that is above the replacement level fertility because the extended life expectancy allows people to grow older. In this case, there will be positive population growth. If the fertility rate is lowered to meet the replacement level fertility, while the life expectancy stays unchanged, then the population will stop growing, and its size will be maintained as it is by definition. Consequently, the aging, in that case, will become a little more rapid than the earlier one, mainly because of the smaller total population. Lastly, when the fertility rate records as the world’s lowest, and the life expectancy is expected to be the world’s highest, then it is logical to contend that the nation will experience the most radical aging in world history. When extreme urbanization (namely, about 91% of the national population resides in urban areas in 2018) is added to such rapid aging, these in tandem specify the case of South Korea.

**Fig. 4:**
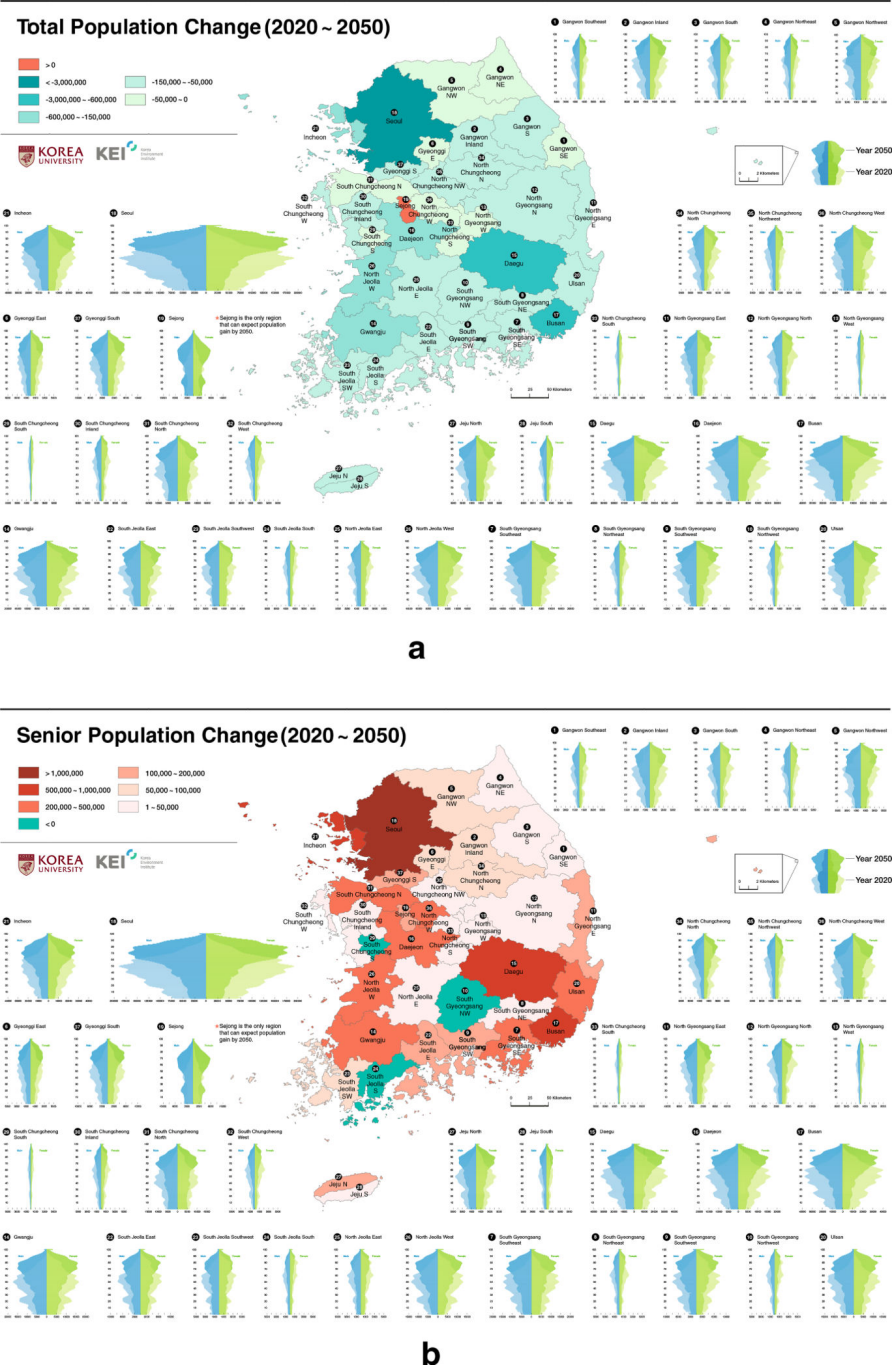
Difference of **a** total and **b** senior population between 2020 and 2050

Given that aging in South Korea is closely associated with its depopulation, it is essential to interpret the aging outcomes with the support of population change maps. Figures 4a and b portray the population changes between 2020 and 2050 with different age categories. The former shows the total population change by taking all ages into account. Sejong is the only region that can expect population gain by 2050, whereas the rest of the regions will have to lose population (Fig. 4a). The latter demonstrates the change of “senior” population whose age is equal to or more than 65 (Fig. 4b). From this map, it is apparent that the three regions (namely, South Chungcheong South, South Gyeongsang Northwest, and South Jeolla South) will experience a decrease in senior population size, unlike the rest. Such drops signify that these three rural and remote regions are even losing senior population, in addition to losing their total population (Figs. 4a and b). In contrast, the rest of the nation will gain enormous senior populations. Sejong will likely experience the most radical aging between 2020 and 2050.

It is critical to note that aging processes differ over regions; thus, plans and policies with respect to mitigating the population aging must take such regional differences into account. For instance, promoting fertility—by introducing governmental subsidies and supports—has been regarded as a significant method in fighting against the nationwide aging and depopulation in South Korea. However, such a method is not likely to work in the rural and remote regions because these areas have already lost their population momentums. In other words, those regions’ aging is already so severe, there are not enough females in the population who can give birth to children to begin with; hence, any governmental subsidies that are geared towards promoting fertility cannot be effective. Given this context, mitigating the extreme urban and migration transition of South Korea, which is still on-going, is more urgent in terms of easing the aging in the rural and remote areas. However, the South Korean government does not seem to even acknowledge the fact that the nation’s radical age transition and depopulation is strongly associated with migration and urban transitions (Presidential Committee on Aging Society and Population Policy, & Ministry of Health and Welfare 2019). If the urbanization migration pattern is reversed—which is, of course, an ideal situation—the reversion can bring fertile female populations to the rural and remote areas. Then finally, a series of plans to promote fertility proposed by the South Korean government may actually work.

Easing the nation’s extreme urbanization is also vital for the metropolitan areas to cope with their severe aging. Because it is clear that more migration to the urban regions will eventually result in a senior population growing more rapidly than before. Within a decade, most baby boomers (who were born between 1955 and 1963, after the Korean War) will retire. Their retirement will not only worsen their own individual financial situations due to their reduced income but also will aggravate the financial situation of the local governments they live in, simply because such retirees cannot pay much tax. By 2050, most echo boomers (offspring of the baby boomers who were born between 1979 and 1985) will, too, retire, and apparently, impacts of such rapid aging are only getting more severe as the year passes by and will affect the nation in diverse ways, mainly pension depletion, increased health care expenditure, limited labor force, vacant residential areas, health problems, etc. (Eun 2008; Park 2019; Park et al. 2007; Tchoe and Nam 2010).

The most urgent health matter would arguably be an immediate shortage of blood supply for medical transfusion (Park et al. 2006), which is also a critical problem in other East Asian countries, such as China due to its rapid aging (Yu et al. 2020). As mentioned earlier, advancement in medicine is a major cause of aging because such progress makes what used to be incurable now curable. However, an unexpected downside is such that advanced medical procedures consume a massive amount of blood, which will be more limited in the future due to the nation’s lowest-low fertility. For example, during operations on a patient to cure his or her cancer or acute illness, it is common to use many units of blood packs for transfusion. Blood is even required for those who have incurable cancer to sustain their lives. Cancer will likely become more frequent as we people age, and it is already the most prominent cause of death in South Korea (Statistics Korea 2018). In short, because there will be so many senior citizens throughout the peninsula, the national demand for blood supply will spike. In contrast, there will be only a limited number of people eligible for supplying the necessary donations of blood. This combination of high in demand and short in supply may degrade the medical treatment and services in South Korea, regardless of its advanced technology.

### 4.2 Methodological Issues

A method-wise comparison is made between the population projection of the present research and that of Statistics Korea (2017). The methods are summarized in Table 2 and differ in terms of sub-model specifics.

Although the two population projections both employ the GLG model for their fertility analyses (Table 2), their underlying assumptions are different. Statistics Korea (2019) assumed that the decline in fertility trend will be reversed in 2021 and afterwards the national fertility will gradually increase by 2051 until the TFR finally reaches 1.68, which is similar to the TFR in 1993 (1.65). This assumption appears not very realistic given the recent TFRs in 2018 and 2019 are 0.98 and 0.92, respectively. Without any revolutionary intervention, it is not likely that the TFR of the following year will be suddenly inverted. In contrast, we applied the ARIMA model to estimate the temporal change of fertility, instead of introducing a strong assumption.

Unlike the Lee-Carter (LC) model and its variants used by Statistics Korea, the HP model has a better parameter structure, and such an aspect is particularly important to the South Korean case because, on the one hand, its population is unique in that it has very high rates of mid-age cancer and adolescent suicide—the 2nd among the OECD (Organisation for Economic Co-Operation and Development) nations (Statistics Korea 2018). Such unusually high rates of cancer and suicide can be factored in adequately by the HP model through the accident mortality term (or “age hump”), hence resulting in a higher goodness-of-fit than do the LC model and its variants (Jeong and Kim 2011; Kim and Jeong 2012; Kim, Lee, and Jeong 2006; Park et al. 2005). On the other hand, the HP model also can ensure a higher goodness-of-fit, given the rapid aging in South Korea. Because the LC model and its variants are designed to model mortality rates of youth and elderly indiscriminately, the LC family is not ideal for a case experiencing rapid aging. In the case of South Korea, it is expected that the mortality of the elderly is likely to become even lower in the future, whereas the mortality of the youth has already reached its lowest level. In this case, the HP model results in a higher goodness-of-fit over the LC family.

Lee et al. (2017) argued that their migrant pool model would perform better than the multiregional model by Statistics Korea (2017) because the model was applied to the 17 provinces in South Korea, and the result turned out more accurate than Feeney’s method. At present, Statistics Korea only serves the province-level population projection and does not provide a more spatially explicit projection. Such a lack of service is likely due to the multiregional model’s limitations when dealing with a larger number of geographical divisions (for example, more than 17). In the presentation made at the Eurostat–UNECE Work Session on Demographic Projections in 2016, researchers from Statistics Korea acknowledged that it was difficult to apply Feeney’s method for smaller and more numerous geographical divisions. This is why they reduced the data dimension by imposing extra restrictions, so that the multiregional model could work for the smaller geographical unit (Kim et al. 2016). The migrant pool model used in this research does not have such limitation by design.

**Fig. 5:**
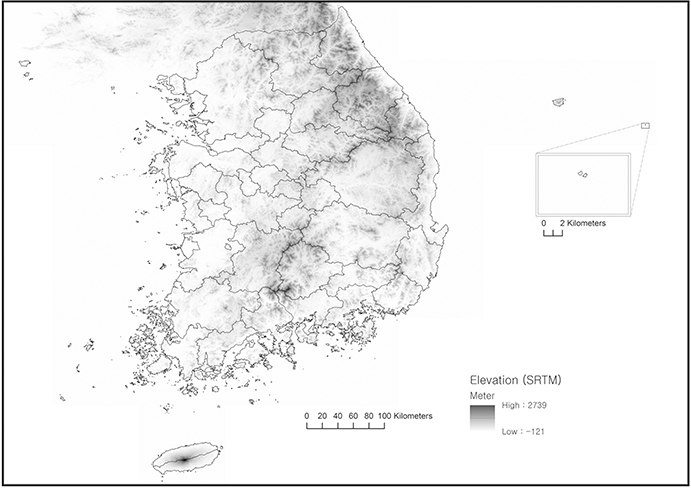
Elevation

The official population projection is important because it is legal. By law, central and local governments must respect the official projection when designing plans and policies. However, the official one does not show a more detailed geographical variation compared to our projection. It is clear that, presently, aging is prevalent in the mountainous areas (Figs. 2 and 5), and such spatial configuration cannot be detected from the official projection. Due to abundant datasets that are freely available, diverse approaches—ranging from a ratio method that is associated with small-area forecasting (Baker et al. 2017) to another component method application based on a more finely specified geographic divisions—can be tested for the Korean case study in the future. Applying spatial methods such as geographically weighted regression is an attractive option when dealing with multiple geographical scales (Evans and Gray 2017; Matthews and Yang 2012; Yang et al. 2013), given South Korea’s extreme urban transition.

## 5 Conclusion

It is logical to conclude that the pace of South Korea’s aging is remarkably fast, even at the global level. From a spatial perspective, two significant demographic megatrends are, and will be, sweeping the nation. At present, the first megatrend dictates the aging of rural and remote regions with the relatively smaller population. The second megatrend will hit the metropolitan areas less than a decade later, and the forthcoming aging of the metropolitan areas will shape the major structure of the national population. To mitigate aging and depopulation in South Korea, taking the extreme and on-going urban transition into account is essential, and such a conclusion could not be made through the official population projection because its spatial resolution is not detailed enough to reveal such sub-national distinctions. Policymakers must come up with a timely plan so that the nation can be prepared for and adapted to the severe aging in South Korea. Solely focusing on the mitigation aspect of population policy, such as promoting fertility, appears unwise as it will take a large amount of time, and yet its success will not be guaranteed, in particular when the urban transition is overlooked.

## Supplementary Material

ESM

## Figures and Tables

**Table 1:** Aging rates in 2020, 2050, their difference (in percent), and future year reaching a super-aged society by region

Region	2020	2050	Difference between 2020 and 2050	Future year reaching super-aged society
Gangwon Southeast	21.92	37.66	15.74	
Gangwon Inland	17.80	39.72	21.92	2023
Gangwon South	22.50	38.88	16.38	
Gangwon Northeast	23.08	39.67	16.59	
Gangwon Northwest	19.32	38.69	19.37	2021
Gyeonggi East	16.56	40.79	24.23	2024
Gyeonggi South	14.12	40.91	26.79	2027
South Gyeongsang Southeast	14.56	38.92	24.36	2025
South Gyeongsang Northeast	29.83	35.07	5.24	
South Gyeongsang Southwest	19.78	40.34	20.56	2021
South Gyeongsang Northwest	33.11	32.81	−0.30	
North Gyeongsang East	19.06	39.38	20.32	2021
North Gyeongsang North	29.46	34.92	5.46	
North Gyeongsang West	30.56	34.83	4.27	
Gwangju Metropolitan	16.97	39.23	22.26	2024
Daegu Metropolitan	16.63	38.43	21.80	2024
Daejeon Metropolitan	16.29	38.85	22.56	2024
Busan Metropolitan	17.51	38.59	21.08	2023
Seoul Megacity	14.29	41.30	27.01	2026
Sejong	9.81	62.26	52.45	2029
Ulsan Metropolitan	13.65	39.90	26.25	2026
Incheon Metropolitan	13.40	42.89	29.49	2026
South Jeolla East	18.70	38.10	19.40	2022
South Jeolla Southwest	22.80	35.87	13.07	
South Jeolla South	33.99	32.75	−1.24	
North Jeolla East	28.31	35.10	6.79	
North Jeolla West	19.26	38.56	19.30	2023
Jeju North	14.39	39.76	25.37	2027
Jeju South	19.77	40.63	20.86	2022
South Chungcheong South	33.46	31.89	−1.57	
South Chungcheong Inland	28.08	34.92	6.84	
South Chungcheong North	13.39	38.23	24.84	2028
South Chungcheong West	29.15	35.13	5.98	
North Chungcheong South	31.95	33.25	1.30	
North Chungcheong North	21.12	37.45	16.33	
North Chungcheong Northwest	21.28	37.20	15.92	
North Chungcheong West	13.11	37.9	24.84	2028

**Table 2:** Model specifications of the two cohort-component methods

	Statistics Korea (2017)	Kim & Kim (the present work)
Fertility	Generalized log gamma distribution model	Generalized log gamma distribution model
Mortality	Li-Lee-Gerland model (Lee-Carter family)	Heligman and Pollard model
Migration	Gross migration (Multiregional model)	Gross migration (Migrant pool model)
Number of Geographical Divisions	17	37
Forecast Horizon	30 years	32 years
