# Supplementary material for: Super Aging in South Korea Unstoppable but Mitigatable: A Sub-National Scale Population Projection for Best Policy Planning
Source: Spat Demogr. Author manuscript; Available in PMC 2021 Jul 1. (PMC8248505; doi:10.1007/s40980-020-00061-8)

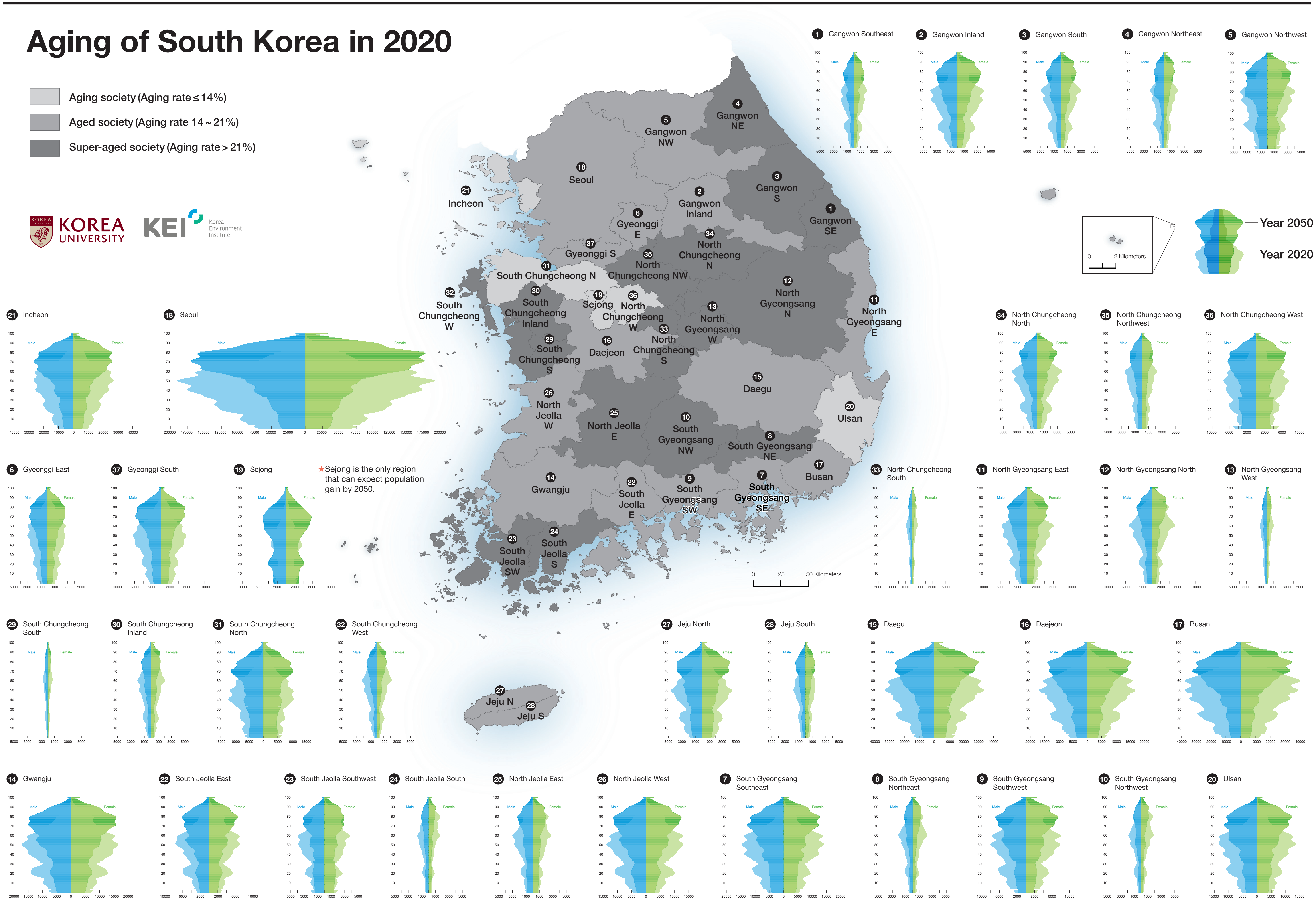
Aging of South Korea in 2020
Aging society (Aging rate ≤ 14%)
Aged society (Aging rate 14 ~ 21%)
Super-aged society (Aging rate > 21%)
KOREA UNIVERSITY
KEI Korea Environment Institute
1 Gangwon Southeast
2 Gangwon Inland
3 Gangwon South
4 Gangwon Northeast
5 Gangwon Northwest
Year 2050
Year 2020
0 2 Kilometers
4 Gangwon NE
5 Gangwon NW
18 Seoul
21 Incheon
3 Gangwon S
2 Gangwon Inland
6 Gyeonggi E
1 Gangwon SE
34 North Chungcheong N
37 Gyeonggi S
35 North Chungcheong NW
31 South Chungcheong N
30 South Chungcheong Inland
32 South Chungcheong W
19 Sejong
36 North Chungcheong W
12 North Gyeongsang N
11 North Gyeongsang E
13 North Gyeongsang W
33 North Chungcheong S
16 Daejeon
29 South Chungcheong S
15 Daegu
26 North Jeolla W
20 Ulsan
25 North Jeolla E
10 South Gyeongsang NW
8 South Gyeongsang NE
17 Busan
14 Gwangju
22 South Jeolla E
9 South Gyeongsang SW
7 South Gyeongsang SE
23 South Jeolla SW
24 South Jeolla S
27 Jeju N
28 Jeju S
0 25 50 Kilometers
21 Incheon
18 Seoul
34 North Chungcheong North
35 North Chungcheong Northwest
36 North Chungcheong West
6 Gyeonggi East
37 Gyeonggi South
19 Sejong
★Sejong is the only region that can expect population gain by 2050.
33 North Chungcheong South
11 North Gyeongsang East
12 North Gyeongsang North
13 North Gyeongsang West
29 South Chungcheong South
30 South Chungcheong Inland
31 South Chungcheong North
32 South Chungcheong West
27 Jeju North
28 Jeju South
15 Daegu
16 Daejeon
17 Busan
14 Gwangju
22 South Jeolla East
23 South Jeolla Southwest
24 South Jeolla South
25 North Jeolla East
26 North Jeolla West
7 South Gyeongsang Southeast
8 South Gyeongsang Northeast
9 South Gyeongsang Southwest
10 South Gyeongsang Northwest
20 Ulsan
Male
Female

Total Population Change (2020 ~ 2050)

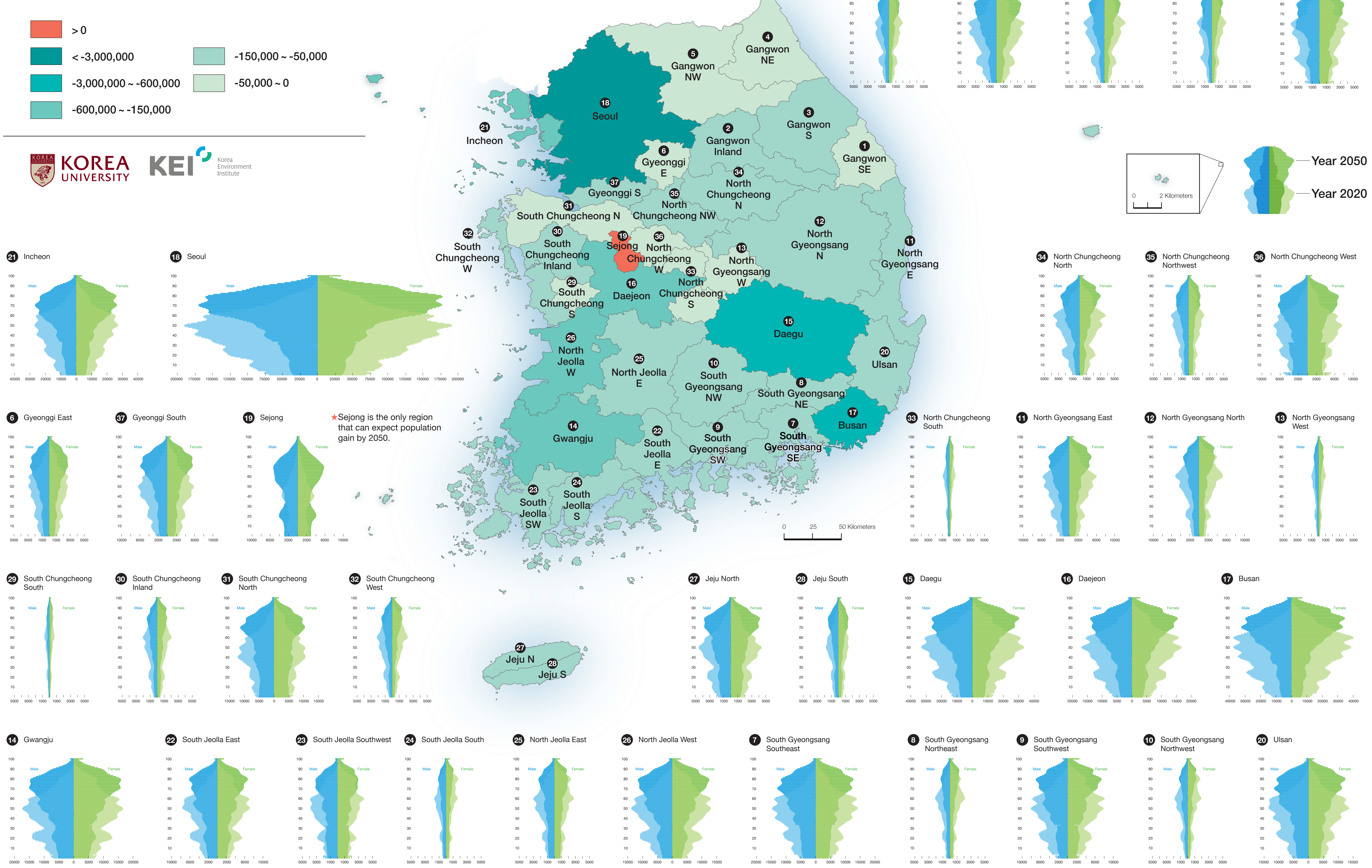

Senior Population Change (2020 ~ 2050)

Colour	Range
dark red	> 1,000,000
red	500,000 ~ 1,000,000
salmon	200,000 ~ 500,000
teal	< 0
light salmon	100,000 ~ 200,000
pale pink	50,000 ~ 100,000
very pale pink	1 ~ 50,000

KOREA UNIVERSITY

KEI Korea Environment Institute

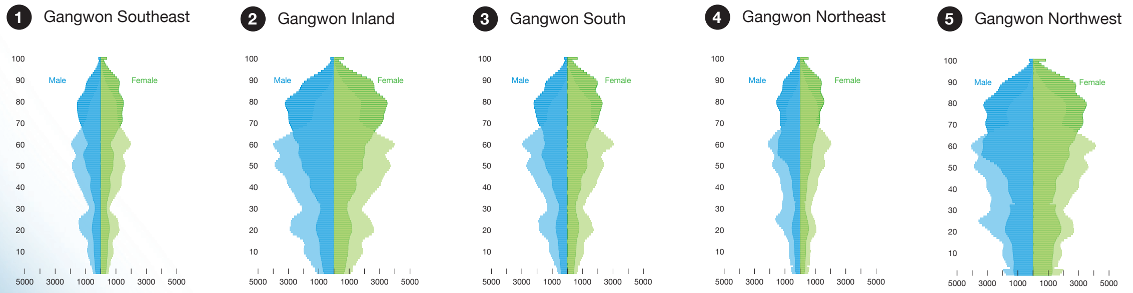

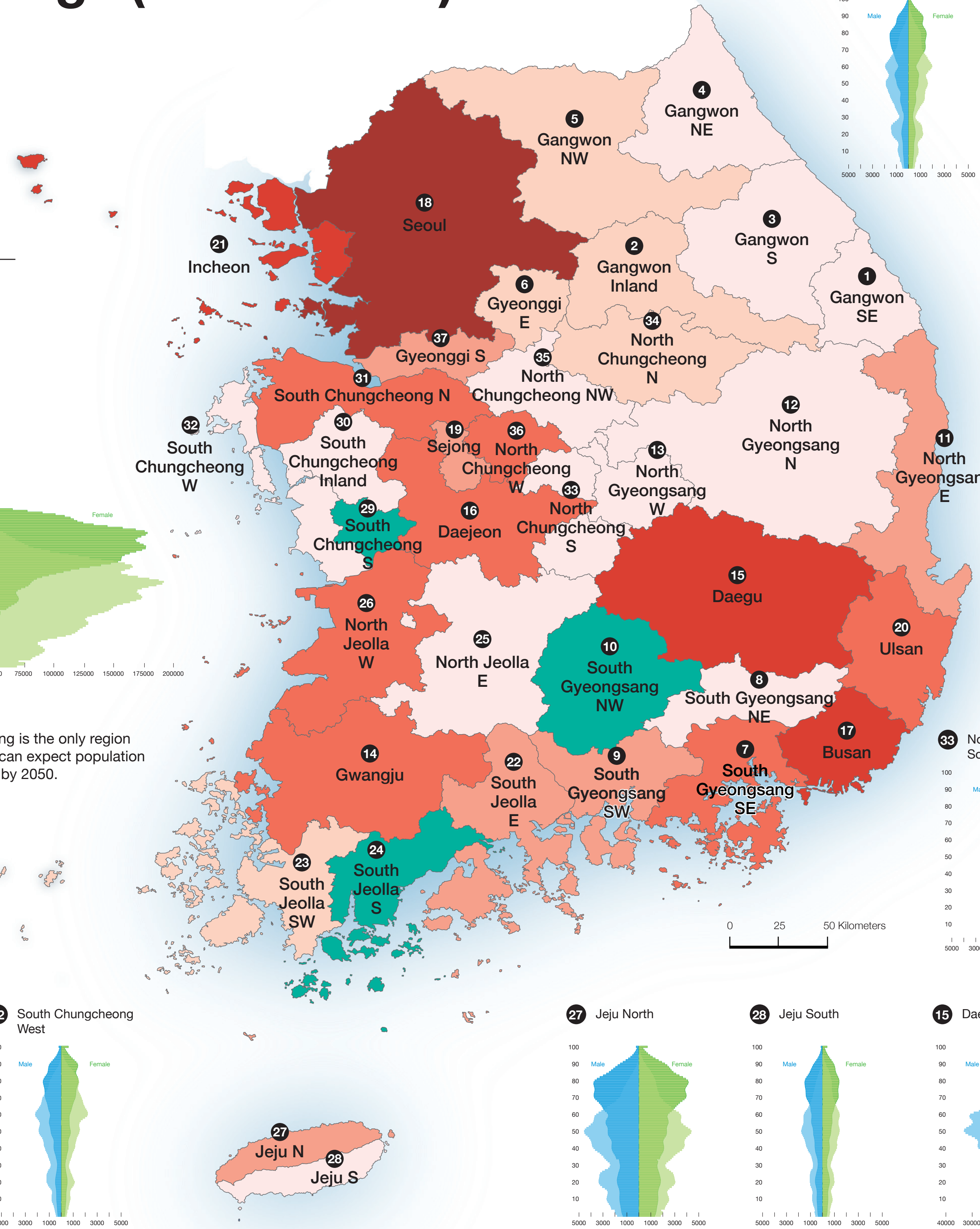

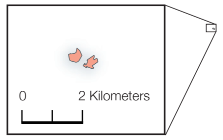

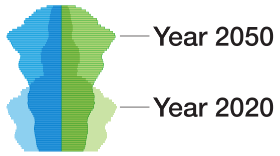

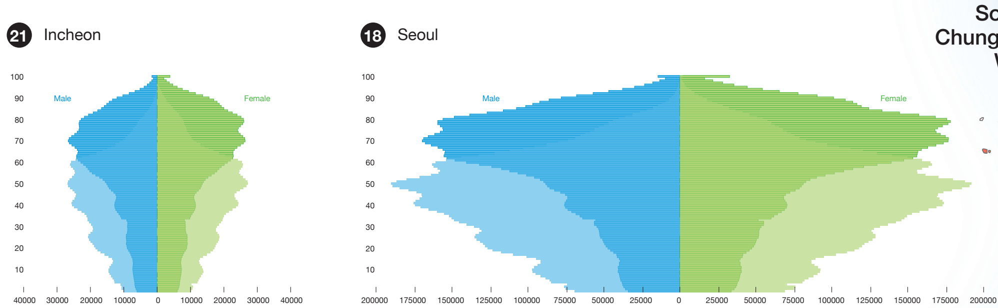

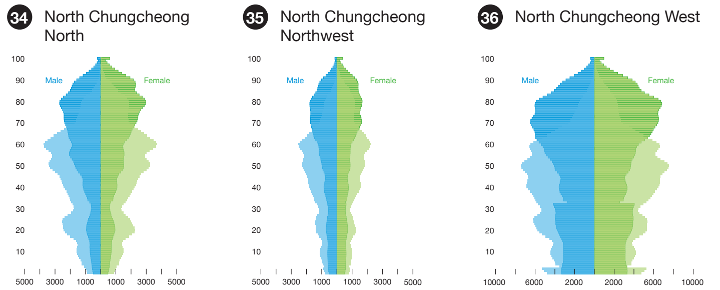

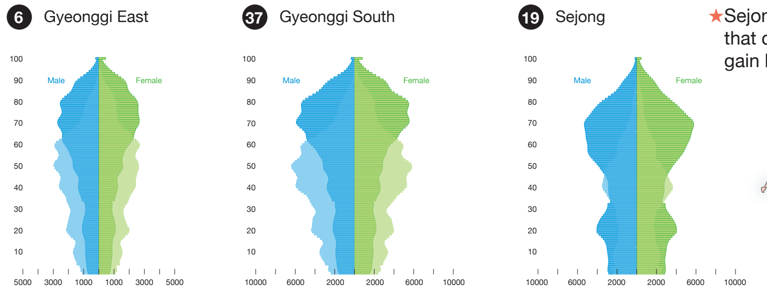

★Sejong is the only region that can expect population gain by 2050.

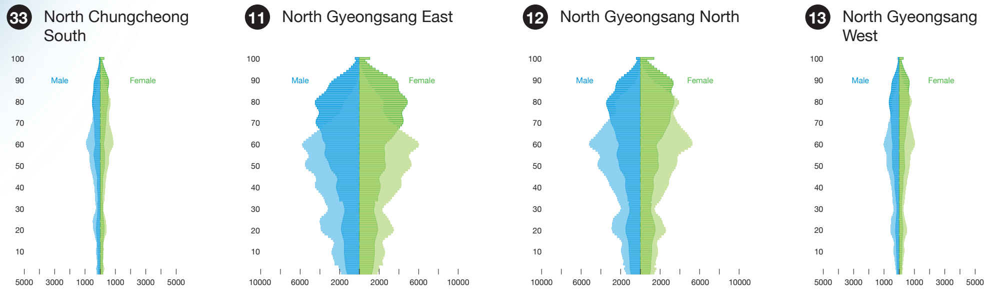

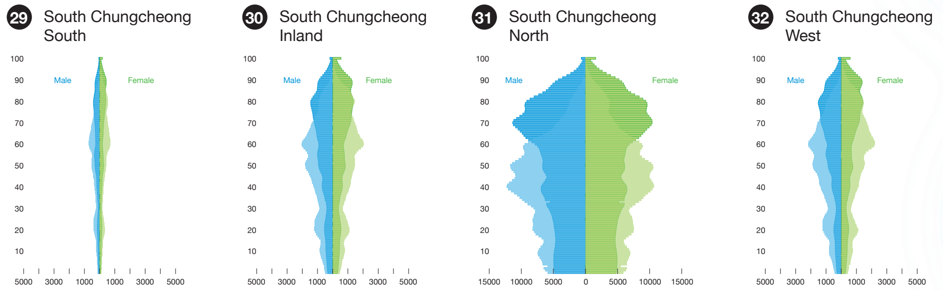

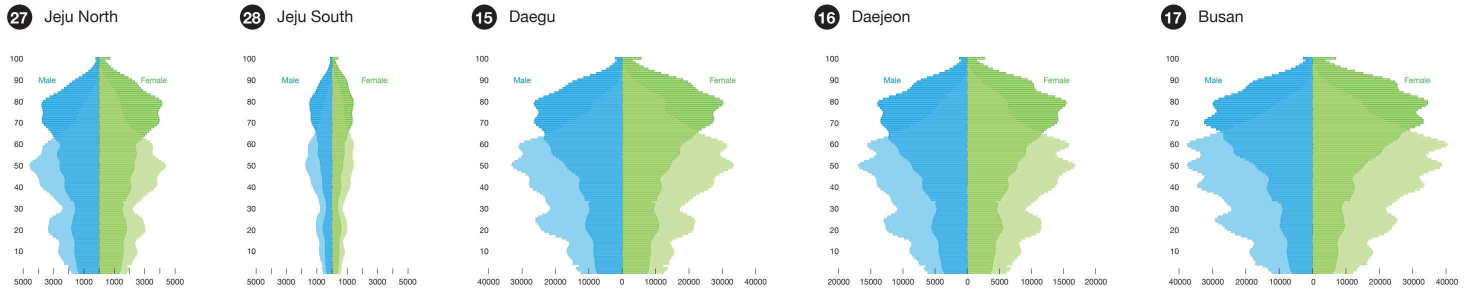

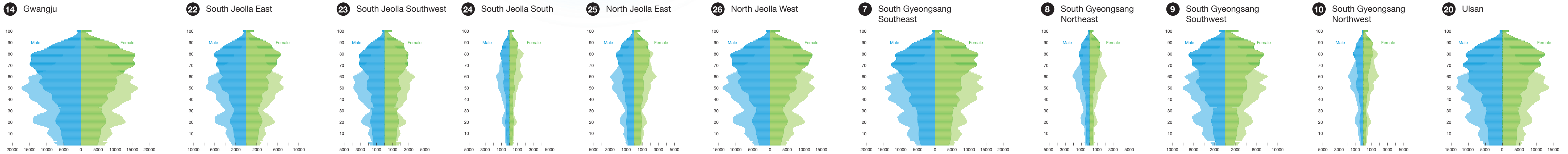

Supplement: ESM [file NIHMS1610115-supplement-ESM.pdf]
